# In Vivo Wound Healing Potential and Molecular Pathways of Amniotic Fluid and Moringa Olifera-Loaded Nanoclay Films

**DOI:** 10.3390/molecules29030729

**Published:** 2024-02-05

**Authors:** Akram Ashames, Munaza Ijaz, Manal Buabeid, Haya Yasin, Sidra Yaseen, Richie R. Bhandare, Ghulam Murtaza

**Affiliations:** 1 College of Pharmacy and Health Sciences, Ajman University, Ajman P.O. Box 346, United Arab Emirates; h.yasin@ajman.ac.ae (H.Y.); r.bhandareh@ajman.ac.ae (R.R.B.); 2 Medical and Bio-Allied Health Sciences Research Centre, Ajman University, Ajman P.O. Box 346, United Arab Emirates; 3 Department of Microbiology, University of Central Punjab, Lahore 54000, Pakistan; munazza3murtaza@gmail.com; 4 Department of Pharmacy, Fatima College of Health Sciences, Abu Dhabi P.O. Box 3798, United Arab Emirates; manal.buabeid@fchs.ac.ae; 5 Department of Pharmacy, COMSATS University Islamabad, Lahore Campus, Lahore 54000, Pakistan; drhsidrarph@gmail.com

**Keywords:** wound healing, inflammation, amniotic membrane fluid, antioxidant, antibacterial, AMF-*Me.mo* nanofilms

## Abstract

Cutaneous wounds pose a significant health burden, affecting millions of individuals annually and placing strain on healthcare systems and society. Nanofilm biomaterials have emerged as promising interfaces between materials and biology, offering potential for various biomedical applications. To explore this potential, our study aimed to assess the wound healing efficacy of amniotic fluid and *Moringa olifera*-loaded nanoclay films by using in vivo models. Additionally, we investigated the antioxidant and antibacterial properties of these films. Using a burn wound healing model on rabbits, both infected and non-infected wounds were treated with the nanoclay films for a duration of twenty-one days on by following protocols approved by the Animal Ethics Committee. We evaluated wound contraction, proinflammatory mediators, and growth factors levels by analyzing blood samples. Histopathological changes and skin integrity were assessed through H&E staining. Statistical analysis was performed using SPSS software (version 2; Chicago, IL, USA) with significance set at *p* < 0.05. Our findings demonstrated a significant dose-dependent increase in wound contraction in the 2%, 4%, and 8% AMF-*Me.mo* treatment groups throughout the study (*p* < 0.001). Moreover, macroscopic analysis revealed comparable effects (*p* > 0.05) between the 8% AMF-*Me.mo* treatment group and the standard treatment. Histopathological examination confirmed the preservation of skin architecture and complete epidermal closure in both infected and non-infected wounds treated with AMF-*Me.mo*-loaded nanofilms. RT-PCR analysis revealed elevated concentrations of matrix metalloproteinases (MMPs) and vascular endothelial growth factor (VEGF), along with decreased levels of tumor necrosis factor-alpha (TNF-α) in AMF-*Me.mo*-loaded nanofilm treatment groups. Additionally, the antimicrobial activity of AMF-*Me.mo*-loaded nanofilms contributed to the decontamination of the wound site, positioning them as potential candidates for effective wound healing. However, further extensive clinical trials-based studies are necessary to confirm these findings.

## 1. Introduction

The skin serves as the primary organ responsible for protecting the body against environmental factors. However, when the integrity of the skin is compromised due to injury or infection, it can lead to severe complications or even mortality [1]. Wounds can be classified into two types: acute and chronic, reflecting the loss of tissue biological integrity [1]. Cutaneous wounds present a silent yet often overlooked burden on the healthcare systems and society, affecting millions of individuals annually [2].

The process of wound healing encompasses multiple sequential stages, including hemostasis, swelling, cellular proliferation, migration, angiogenesis, and extracellular matrix remodeling [3]. The initial phase of wound healing involves the activation of a clotting cascade at the injury site. This is followed by the second phase, characterized by the infiltration and activation of immune cells (neutrophils, lymphocytes, and macrophages). These immune cells play a crucial role in eliminating microorganisms and clearing tissue debris from the wound site. The third phase of wound healing is marked by cellular proliferation and differentiation. This phase primarily involves processes such as epithelization, angiogenesis, and granulation tissue formation [3]. These physiological events contribute to the restoration and regeneration of the damaged tissue during wound healing. Angiogenesis, a vital process in wound repair, involves the invasion of new blood vessels into the wound site. This process includes matrix degradation and the proliferation of endothelial cells, contributing to tissue regeneration. Through angiogenesis, damaged areas are re-vascularized, enabling the delivery of oxygen and essential nutrients to support the healing process. Additionally, angiogenesis plays a role in the closure of epithelial cells, facilitating the restoration of tissue integrity. The final phase of the healing process is referred to as “maturation”, which plays a crucial role in the strengthening and remodeling of the newly formed tissues over a period of several months [4]. During this phase, the extracellular matrix undergoes further restructuring, and the newly generated tissue gradually matures and gains strength. This phase is essential for achieving optimal tissue function and structural integrity in the healed area.

The factors that affect the wound healing process are categorized into local (e.g., proinflammatory cytokines, reactive oxygen species (ROS), non-functionality of proteases, and deficiency of growth) and systemic (chronic pathological conditions including aging, diabetes mellitus, stress, obesity, sickle cell disease, and Cushing syndrome) [5]. Indeed, bacterial infection is a common factor that can significantly impede the wound healing process. Therefore, the timely identification and appropriate management of bacterial infections are crucial for promoting optimal wound healing outcomes. The colonization of “opportunistic bacteria” initiates the formation of an extracellular polymeric substance (EPS) matrix. This matrix provides a scaffold for the bacteria to proliferate in a nutrient-dependent manner, ultimately leading to the formation of a biofilm at the wound site. Following the formation of biofilms, the host immune response becomes insufficient in effectively clearing the infection. In an attempt to combat the biofilm, leukocytes bind to the biofilm and release enzymes that aggravate a prolonged inflammatory stage [6,7]. Studies have demonstrated that a significant proportion of individuals, approximately 10%, succumb to complications arising from the lack of suitable treatment of chronic wounds [8]. Platelet-rich plasma (PRP) is an emerging therapeutic technology that has garnered significant attention in the field of regenerative medicine due to its potential to stimulate and accelerate tissue healing [9]. PRP is derived from the patient’s own blood and contains concentrated numbers of platelets, growth factors, and bioactive proteins. This autologous preparation has shown promising potential in stimulating and accelerating tissue healing processes. The growth factors and other bioactive molecules present in PRP have been reported to promote cell proliferation, angiogenesis, and tissue regeneration, making it an attractive option for various medical applications. As a result, PRP is being explored and utilized in a wide range of fields, including orthopedics, dermatology, dentistry, and wound healing. Platelets play a crucial role in the wound healing process by secreting various cytokines and growth factors such as platelet-derived growth factor (PDGF), epidermal growth factor (EGF), fibroblast growth factor (FGF), insulin-like growth factor (IGF1, IGF2), vascular endothelial growth factor (VEGF), transforming growth factor (TGF-β), and keratinocyte growth factor (KGF) [10].

In the modern era, the wound healing process can be augmented with the use of “natural products”. Studies have investigated the wound healing properties of herbal medicines, which possess various actions such as anti-inflammatory, antibacterial, antioxidant, antibacterial, and pro-collagen synthesis effects [11]. Findings have shown that Curcuma longa (turmeric), Ephedra ciliata, Epis mellifera (honey), Rauwolfia serpentina (snake wood), Centella asiatica (Asian pennywort), Moringa olifera (drumstick tree), Panax ginseng (Ginseng), Azaractica Indica (Neem), and barbadensis (Aloe vera) possess superb wound healing potential and their therapeutic properties may be due to the presence of bioactive phytochemicals such as alkaloids, essential oils, flavonoids, tannins, terpenoids, saponins, and phenolics [12]. Indeed, numerous in vitro and in vivo studies have demonstrated the promising role of vitamins (e.g., tocopherol, tocotrienol) and trace elements (e.g., zinc) in wound repairing due to their bone resorption and immune modulating properties [13]. Tocopherol and tocotrienol, both forms of vitamin E, have been shown to promote collagen synthesis, reduce inflammation, and enhance antioxidant defense mechanisms, thereby facilitating tissue regeneration. Similarly, zinc has been found to play a crucial role in various stages of wound healing, including cellular proliferation, collagen synthesis, and immune function modulation. Despite significant advances in clinical consideration there is a dire need to develop novel therapies for wound healing purposes. The clinical field is quickly progressing towards the advancement of accelerated treatments that can decrease morbidity and provide good quality of life to patients [14]. Regenerative medications represent an emerging interdisciplinary domain in biomedical field that aims to re-establish, recover, and replace harmed tissues and cells. For instance, amniotic membrane fluid (AMF) is an attractive moiety for applications in regenerative medications due to its proliferation capacity, immune boosting activity, and multipotent nature. Furthermore, AMF has the ability to liberate anti-inflammatory cytokines inside the body and thus can modulate inflammatory responses efficiently [15].

In drug delivery systems, systemic and oral routes have been traditionally employed for drug administration. However, the systemic route often poses challenges such as toxicity concerns and less predictable drug delivery to the target tissue. As a result, researchers have increasingly focused on localized drug delivery for wound healing [16]. Wound dressing not only heals wounds but also helps in the regeneration of epidermal tissue and protects wounds from invading microorganisms [17]. In ancient periods, wound dressings with the direct use of crude plant extracts were harmful as they might contain synthetic substances or microorganisms, which might cause infection. Thus, this concept provoked the development of dressings comprising powerful anti-microbial moiety and highly potent herbal medicines [18,19]. The understanding of wound healing took a significant shift in 1962 when the conventional belief that keeping wounds dry would promote rapid recovery was challenged. This change in perception was brought about by the groundbreaking work of Winter, who pioneered the development of “moist wound films.” and revealed that wound healing can be faster in a wet environment as it supports rapid re-epithelization and cellular growth [20]. The present study was designed to evaluate the wound healing potential of amniotic fluid and Moringa olifera-loaded nanoclay films by using in vivo models. Additionally, this study investigated the antioxidant and antibacterial properties of these films The objective was to explore the effectiveness of these novel nanoclay films in promoting wound healing processes and their potential for mitigating oxidative stress and combating bacterial infection in the wound area, and the potential of nanomaterial-based approaches for improved wound care.

## 2. Results

### 2.1. Physical Analysis of Films

The thicknesses of three nanoclay-containing AMF-*Me.mo*-loaded nanofilms were measured to be 0.5 ± 0.02 (2% AMF-*Me.mo*), 0.8 ± 0.01 (4% AMF-*Me.mo*), and 0.6 ± 0.01 (8% 0.6 ± 0.01) milliliters, respectively, while the 2%, 4%, and 8% nanofilms showed moisture contents at 40.5 ± 0.09, 56.4 ± 0.12, and 33.6 ± 0.05, respectively. The increase in water absorption promotes the crystallinity structure and decreases the tensile strength of the matrices. Table 1 exhibits the order of crystallinity in descending order as 2% AMF-*Me.mo* < 8% AMF-*Me.mo* < 4% AMF-*Me.mo* nanofilms (Table 1).

### 2.2. Antioxidant Activity

According to a DPPH assay, the free radical scavenging activity of AMF-*Mo.me* nanofilms increases in a concentration-dependent manner (Table 2). The lower value of IC50 indicates a strong antioxidant potential. The ascorbic acid showed a strong free radical scavenging activity.

### 2.3. Antibacterial Activity

**Figure 1:**
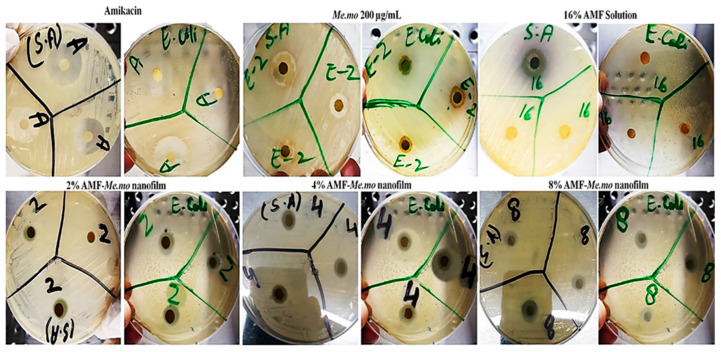
Antibacterial effect of amikacin, *Moringa olifera* extract, and 2%, 4%, and 8% AMF solution against *Staphylococcus aureus* and *Escherichia coli* bacteria.

Table 3 presents the effects of AMF-*Me.mo*, AMF solution, *Me.mo*, and amikacin in terms of zones of inhibition. The formulations of 2%, 4%, and 8% AMF-Me.mo exhibited significant inhibitory effects against both bacterial strains, as depicted in Figure 1. However, the AMF solution alone displayed no activity against *E. coli* (Gram-negative), while demonstrating positive inhibitory action against *S. aureus* (Gram-positive). Amikacin and the methanolic extract of *Moringa olifera* exhibited more pronounced antibacterial activity against both Gram-positive and Gram-negative bacterial strains compared with all treatment formulations. These findings suggest the potential of amikacin and the methanolic extract of *Moringa olifera* as potent antibacterial agents.

### 2.4. Wound Healing Activity

**Figure 2:**
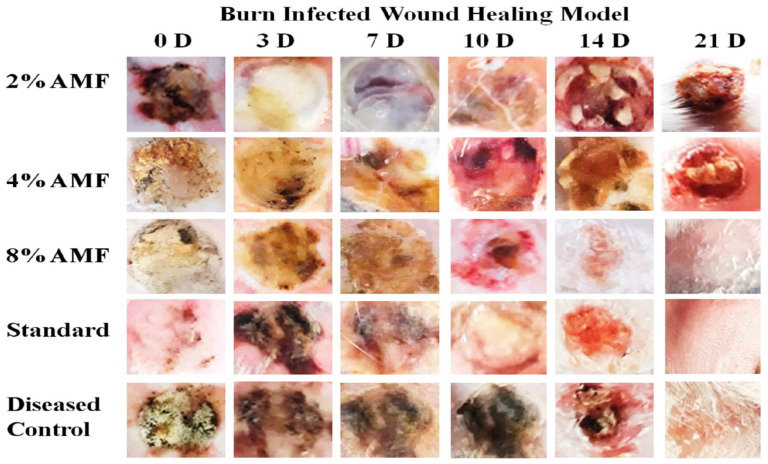
Macroscopic analysis of burn infected wound healing model.

**Figure 3:**
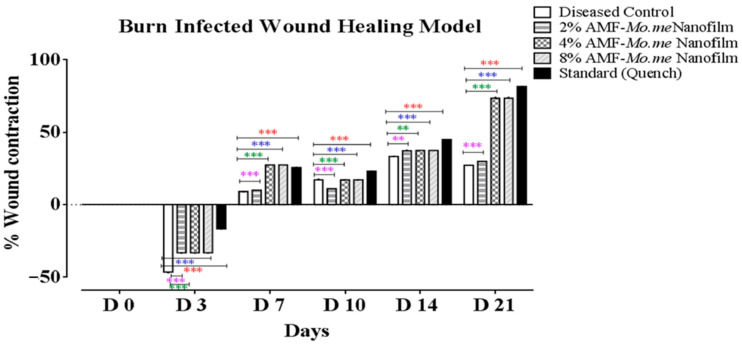
Graphical representation of percent wound contraction in infected wounds. Values shown are Mean ± SEM (*n* = 4). Where ** = *p* ˂ 0.01, *** = *p* ˂ 0.001. Violet, green, and blue color stars show comparison of 2%, 4%, and 8% nanoclay-based AMF-*Moringa*-loaded nanofilm treatment groups with diseased control. While red color stars show comparison of standard (Quench^®^) with diseased control.

Table 4 presents the effect of AMF-*Me.mo* on the infected wounds in terms of percentage closure. On the third day, the percent wound contraction was started in the AMF-*Me.mo* nanofilms and standard treatment groups efficiently. Negative signs showed an inflammatory response. On the seventh day, percentage contraction was significantly (*p* ˂ 0.001) increased in the 2%, 4%, and 8% AMF-*Me.mo*-loaded nanofilms treatment groups in a dose dependent manner compared with the control, while the 8% AMF-*Me.mo*-loaded nanofilm showed comparable (*p* > 0.05) wound healing effects with the standard. On the tenth day, the observed increase in the percentage contraction value of the 2% and 4% AMF-*Me.mo* treatment groups was found to be comparable to that of the diseased control group. This suggests that these lower dose levels exhibited slightly less pronounced healing effects compared with the higher dose level (8% AMF-*Me.mo*). These findings imply that the effectiveness of the AMF-Me.mo treatment may be dose-dependent, with the higher concentration showing more significant healing effects. Further analysis and comparison of the different dose levels will provide a more comprehensive understanding of the optimal concentration for achieving optimal wound healing outcomes. However, the 8% AMF-*Me.mo* treatment group showed significant (*p* ˂ 0.001) elevation in % contraction and thus showed remarkable healing effects on wounds, like the standard treatment. On the 21st day, the percent wound contraction values of both the AMF-*Me.mo* and Quench^®^ cream treatment groups exhibited a significant increase (*p* < 0.001) when compared with the diseased control group. This significant increase indicates that both the AMF-*Me.mo* and Quench^®^ cream treatments possess favorable healing properties. Figure 2 and Figure 3 visually demonstrated the notable improvement in wound healing in the treatment groups compared with the diseased control group. These results highlight the potential efficacy of the AMF-*Me.mo* and Quench^®^ cream treatments in promoting the healing process and suggest their potential application as therapeutic interventions for wound healing.

**Figure 4:**
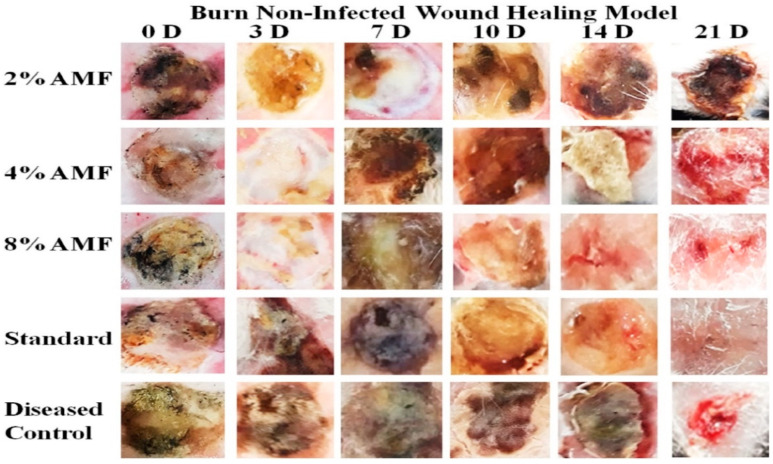
Macroscopic analysis of burn non-infected wound healing model.

**Figure 5:**
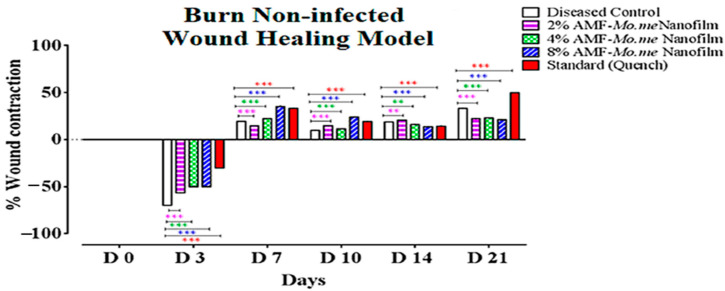
Graphical representation of percent wound contraction in non-infected wounds. Values shown are Mean ± SEM (*n* = 4). Where ** = *p* ˂ 0.01, *** = *p* ˂ 0.001. Violet, green, and blue color stars show comparison of 2%, 4%, and 8% nanoclay-based AMF-*Moringa*-loaded nanofilms treatment groups with diseased control, while red color stars show comparison of standard (Quench^®^) with diseased control, respectively.

Table 5 shows the effect of AMF-*Me.mo* on non-infected wounds. On the third day, percent wound contraction was observed to be increased in the standard group and the 2%, 4%, and 8% AMF-*Me.mo* treatment groups significantly (*p* ˂ 0.001) compared with the diseased control. A negative sign indicates inflammation. On the seventh day, percentage contraction was significantly (*p* ˂ 0.01) increased in the 2%, 4%, and 8% AMF-*Me.mo*-loaded nanofilms and the Quench^®^ cream-treated groups as compared with the control. On the 10th day, the values of the percent wound contraction were raised efficiently, depicting a dose dependent healing response of the 2%, 4%, and 8% AMF-*Me.mo* treatment groups. In addition, the standard and 8% AMF-*Me.mo*-treated animals showed comparable effects (*p* ˂ 0.001). On the 14th day, percentage contraction was significantly (*p* ˂ 0.01) increased in the 2%, 4%, and 8% AMF-*Me.mo*-loaded nanofilm treatment groups compared with the diseased control. On the 21st day, the AMF-*Me.mo* treatment groups (2%, 4%, and 8%) exhibited statistically significant (*p* ˂ 0.001) wound healing potential as compared with the diseased control group. Moreover, the Quench^®^ cream- and the 8% AMF-*Me.mo*-treated animals produced a comparable reduction in inflammation at the wound site, thus demonstrating notable wound healing effects throughout the study (Figure 4 and Figure 5).

**Figure 6:**
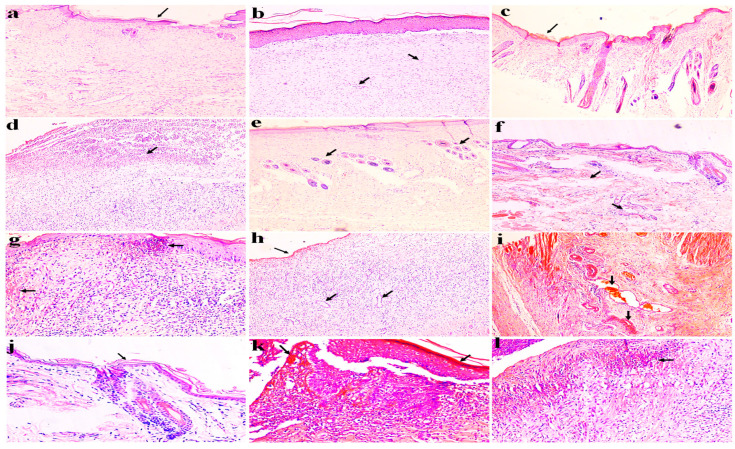
Histopathological analysis of infected burn wound healing animals treated with Quench^®^ (**a**,**b**) and 8% (**c**,**d**), 4% (**e**–**g**), and 2% (**h**,**i**) AMF-*Me.mo*-loaded nanocomposites and diseased control (**j**–**l**) group, respectively. The arrows in this figure represent the above-mentioned processes (granulation tissue formation, occurrence of blood vessel growth, development of fibroblasts, scab formation, hypertrophy, atrophy, and the infiltration of macrophages and neutrophils in various images) as indicated by arrows.

Figure 6 shows the histopathological analysis of infected burn wound healing animals treated with Quench^®^ (a,b) and 8% (c,d), 4% (e–g), and 2% (h,i) AMF-*Me.mo*-loaded nanocomposites and the diseased control (j–l) group, respectively. Apparently, wounds treated with Quench^®^ cream (standard) and the 8% AMF-*Me.mo*-loaded nanocomposites showed intact skin structure with complete re-epithelization. However, the mild recruitment of inflammatory cells (neutrophils and macrophages) has been examined under a microscope. In the 4% and 2% AMF-*Me.mo* treatment groups, granulation tissue formation was analyzed and indicated an ongoing wound healing process. Moreover, the occurrence of blood vessel growth and the development of fibroblasts as well as scab formation in animals’ skin was observed to be associated with the continuous application of AMF-*Me.mo* nanocomposites. Despite that, atrophy was observed in animals treated with 2% and 4% AMF-*Me.mo* nanofilms. In the diseased control group, hypertrophy, atrophy, and the infiltration of macrophages and neutrophils was clearly observed.

### 2.5. Measurement of TNF-α and TGF-ß1 and MMP Levels

Table 6 represents the activation of vascular endothelial growth factor and matrix metalloproteinases concentrations with the application of AMF-*Moringa*-loaded nanofilms. The levels of both mediators were observed to be significantly increased in a dose-dependent manner compared with that of the control group. However, the concentration of tumor necrosis-alpha (pro-inflammatory mediator) was observed to be lower in the standard > 8% > 4% > 2% AMF-*Moringa* treatment groups as compared with the diseased control group.

## 3. Discussion

Nanofilm biomaterials act as an interface between material and biology and offer great promise toward several new and emerging biomedical applications [21]. To the best of our knowledge, this is the first study that ascribed the wound healing effect of pectin-sericin-based AMF-*Me.mo*-loaded nanofilms. The antibacterial and antioxidant activity of three AMF-*Me.mo* nanofilms were also investigated. The nanofilms were fabricated with an adequate control by tuning the reaction’s parameters in the current research work [22]. Polymers act as binding agents as well as enhance the viscosity of formulation, while the addition of glycerol provides sufficient film thickness. Thin films allow for the easy administration of the drug and are economical to use. The presence of sufficient water content in the formulation maintains the integrity, texture, and mechanical properties of the nanocomposites [23]. In the present work, the findings suggest significant variations in the thickness of nanofilms from a range of 0.5 to 0.8 mm, indicating changes in the drug releasing behavior and physicochemical properties of the nanofilms. These peculiar characteristics make them suitable for different applications, i.e., pharmaceutical drug delivery, biological reactors, and so on [24]. In addition, biocompatibility, flexibility, and the possibility to carry drugs are some of the most interesting features that nanofilms can exploit.

Different antioxidant substances used in wound dressing have shown positive effects on the wound healing process through the regulation of oxidative stress. Studies have shown that potent DPPH free radical scavenging activity might be ascribed due to its “electron donating or hydrogen ion neutralization” property [25,26]. The results of the present research showed the moderate antioxidant activity of three AMF-*Me.mo* nanofilms, suggesting that plant extract and amniotic membrane fluid are responsible for detaining levels of reactive oxygen species. In addition, it is well documented that flavonoids and phenolic compounds of *Moringa olifera* contribute directly to antioxidative action as described in earlier reports [27,28]. Thus, one of the reasons behind the development of AMF-*Me.mo*-based nanofilm systems is their good antioxidant activity.

The imbalance between the local pathological factors and immune system integrity at the wound site may encourage the colonization of both Gram-positive and Gram-negative bacteria. Among all, the most detected bacterial strain at the site of injury are *Staphylococcus aureus* and *Escherichia coli* [29]. Therefore, the antibacterial activity of biosynthesized AMF-*Me.mo*-loaded nanofilms was evaluated against these clinical pathogens (*S. aureus and E. coli*) using disc diffusion assay. A clear zone of inhibition (ZOI) was observed around discs loaded with 2%, 4%, and 8% AMF-*Me.mo* nanofilms, a single AMF solution, and a methanolic extract of *Moringa olifera* and amikacin. The findings clearly depicted that AMF-*Me.mo*-loaded nanofilms showed a considerable inhibitory pattern against both bacterial strains in a concentration-dependent manner (2% < 4% and < 8%). Moreover, simple AMF and *Me.mo* extract also exhibited significant antimicrobial effects like the nanofilms. Overall, the test samples showed comparable effects (*p* < 0.001) with amikacin, as detailed in the previous studies.

Wounds provide a conducive environment for a variety of microorganism’s growth (i.e., *S. aureus*, E. coli, Clostridium tetani, and enterococcus), which ultimately delays healing and causes a more pronounced acute inflammatory reaction [30]. In the current study, the evaluation of AMF-*Me.mo* extract in clinically created infected wounds showed dose-dependent inhibitory activity against pathogenic microorganisms including *S. aureus* and *E. coli*. Moreover, the antimicrobial potential of the methanolic extract of *Moringa olifera* and amniotic membrane fluid may partly contribute to the wound healing effect by eliminating infection and thus allowing the initiation of natural tissue repair processes. Moreover, the results of non-infected wounds suggested that the application of AMF-*Me.mo* nanofilms may play a useful role in accelerating the healing of wounds. This can be supported by the fact that a greater reduction in the rate of wound contraction demonstrates a better efficacy of AMF-*Me.mo* nanofilms and, consequently, wounds close at a faster rate.

Histopathology examination has always been considered as a “gold standard” parameter in diagnosing certain infectious, degenerative, or neoplastic diseases in humans and animals. In the present study, the application of AMF-*Me.mo* treatment on animals showed a dose-dependent integrity of skin architecture with complete epidermal closure in both infected and non-infected wounds. The infected wounds showed a faster maturation of granulation tissue, thus represent a slightly more rapid healing of wounds compared with non-infected wounds. In addition, healing was observed to be slow in the 4% and 2% treatment groups, which may be due to the addition of lower concentrations of amniotic fluid in their formulation. Hence, AMF-*Me.mo* nanofilms were found to be effective for both bacterial and non-bacterial infections occurred at the wound sites. Metalloproteinase is a group of matrix proteins that play a crucial role in tissue remodeling and re-epithelization [31]. Growth factors (e.g., VEGF, TGF-β, and so on) have chemotactic activities that improve the healing of wounds by attracting inflammatory cells and fibroblasts. Moreover, they act as mitogens and enhance cellular proliferation at the injury site [32]. In the present study, the raised concentrations of MMPs and VEGF are attributed to the supportive role of AMF-*Me.mo*-loaded nanofilms in the natural healing process by modulating blood vessel growth and the crucial turnover of cells such as collagen and elastin. Moreover, the inhibitory effect of nanofilms on TNF-α levels is a critical event in reversing the worsening of sounds by promoting the formation of an extracellular matrix. Overall, AMF-*Me.mo*-loaded nanofilms possess good wound healing potential and can be applicable as good candidature to the treatment of impaired wounds.

## 4. Materials and Methods

### 4.1. Materials

All chemicals used in this study were of analytical grade and purchased from different distributors: pectin and sericin (Sigma Aldrich^®^, St Louis, MO, USA), nanoclay (Sigma Aldrich^®^, USA), nutrient agar (CM 003, Oxoid, Basingstoke, UK), nutrient broth (HiMedia, San Diego, CA, USA), MH nutrient (HiMedia, San Diego, CA, USA), glycerol (Sigma Aldrich^®^), 2,2-diphenyl-1-picrylhydrazyl (DPPH), methanol/ethanol (Sigma Aldrich^®^). *Moringa olifera* extract was prepared in the Pharmacognosy lab. Xylazine^®^, ketamine^®^, and amikacin^®^ were purchased through commercial sources.

### 4.2. Collection of Plant Material

*Moringa olifera* leaves were collected from Benessere Health Company, Multan, Pakistan, and authenticated by an expert botanist, Dr. Altaf Hussain Dasti from the Department of Botany, Bahauddin Zakariya University Multan, Pakistan. The voucher specimen was submitted to the university herbarium (Voucher No. 78X/20) for further experimentations.

### 4.3. Extract Preparation 

Leaves of *Moringa olifera* were washed with water and dried under air shade for a period of two weeks. After cleaning, leaves were ground and fine powder was macerated with methanol in an airtight container for three days. The container was shaken periodically on each day and the marc was separated out through a filtration technique (Whatman filter paper # 01) [33]. The moisture was evaporated by using a rotary evaporator to make it solvent-free. The extract was preserved in refrigerator (−4 °C) for further use and labelled as *Mo.me.*

### 4.4. Isolation and Preparation of Amniotic Membrane Fluid 

Initially, freshly dissected placenta was placed at 4 °C for 24 h. Then, amniotic membrane was separated from chorion membrane and shining gel-like semitransparent membrane was washed with balance sodium solution thrice. Then, 220 mg of amnion plus 22 mg of pepsin was taken in sterile tube, and hydrochloric acid was added and allowed digestion for a few hours. Finally, solution was centrifuged at 4500 rpm for a certain period of time and supernatant was removed [34]. The solution was labelled as AMF and stored at −80 °C for further use.

### 4.5. Preparation of AMF- and Moringa olifera-Loaded Nanocomposites

To prepare AMF- and *Mo.me*-loaded nanocomposite combinations, pectin and sericin were used. A specific concentration of both polymers was taken, dissolved in distilled water, and heated. Then, polymers were mixed and glycerol was added in mixture to promote film flexibility. Then, a certain amount of *Moringa olifera*, amniotic fluid, and nanoclay was added in polymeric solution and poured in petri plates to obtain thin films. Plates were subjected to dryness in an oven (37 °C) for 2–3 days (Table 7) [35], while films of control were also prepared with a similar method. After 72 h, AMF- and *Mo.me*-loaded nanocomposites were separated out from plates, wrapped in aluminum foil, and stored.

### 4.6. Physical Analysis of Films

#### 4.6.1. Films Thickness

AMF- and *Moringa olifera*-loaded nanocomposites’ thickness were measured with the help of a vernier caliper. Reading was obtained from three different points of each film and calculated average was taken as the whole thickness of that specific film [36].

#### 4.6.2. Moisture Content

The moisture content (MC) of AMF- and *Moringa olifera*-loaded nanocomposites was analyzed with a well-reported method. One uniform segment (1 cm × 1 cm) of each film were cut, weighed, and dried in oven (105 °C) [36]. On the next day, weight of film pieces was measured again and MC was calculated through following formula:

Moisture Content=Wd−Wf/Wd×100 

where *Wd* = initial weight of film segment and *Wf* = Final film weight of film.

### 4.7. In Vitro Studies 

#### 4.7.1. Antibacterial Studies 

Disc diffusion method was adopted to analyze the antibacterial activity of AMF- and *Moringa olifera*-loaded nanocomposites. The composites were tested against *Escherichia coli* (Gram − ve) and *Staphylococcus aureus* (Gram + ve) bacteria. Initially, bacterial strains were activated in liquid lysogeny broth media at 37 °C and incubated for 24 h. Then, swabbing of bacterial solution was performed on solidified nutrient agar-containing plates. The discs of AMF solution (2%, 4%, and 8%), *Mo.me* (200 µg/mL), and amikacin were set on plate. In addition, paper discs, dipped in *Mo.me* and AMF, were also placed on respective Petri plates in triplicates and incubated at 37 °C for 12 h. The zone of inhibition was measured and each sample was recorded separately [37].

#### 4.7.2. Antioxidant Activity through 2,2-Diphenyl-1-Picrylhydrazyl (DPPH) Assay

To assess the antioxidant activity of AMF- and *Moringa olifera*-loaded nanocomposite films, a DPPH assay was performed. A uniform piece (100 mg) of each film was cut and immersed in DPPH solution (0.01 mM). Another group was set as blank containing DPPH solution only. Then, plate was placed at dark place for half an hour and absorbance was determined at 517 nm until 8 h [37]. Antioxidant activity was calculated through following formula:

$$Antioxidant\;\mathrm{potential}=\left(A0-\mathrm{At}\right)/A0\times 100\;$$

where A0 and At were absorbance of blank and sample solution, respectively.

### 4.8. In Vivo Studies 

#### 4.8.1. Burn Wound Model

Five healthy rabbits weighing 200−250 g at 6 weeks old were purchased and kept in the animal house of Department of Pharmacy, COMSATS University Islamabad, Lahore Campus, Lahore, Pakistan. Animals were housed for 1 week prior to experimentation and food as well as water supply was ensured. 

#### 4.8.2. Wound Healing Activity

Animals were divided into five groups as Group-1: Diseased Control; Group-2: 2% AMF-Moringa-loaded nanocomposites; Group-3: 4% AMF-Moringa-loaded nanocomposites; Group-4: 8% AMF-Moringa-loaded nanocomposites; and Group-5: Quench cream^®^. Animal’s hair was removed from dorsal surface and skin was cleaned with ethanol. Subsequently, animals were anesthetized using ketamine–xylazine combination. Two full-thickness wounds of 1.5 cm were created with the help of metal strip on rats’ back. One wound was labelled as infected (containing mixture of *Staphylococcus aureus* and *Escherichia coli* bacterial straining) and the other as noninfected. Then, an equal size of each film sample was cut and the wound site was covered with them. The wound area was measured with vernier caliper on 0, 3, 7, 10, 14, and 21 days. Additionally, digital photographs were also taken on respective days of measurement [37]. The percentage wound contraction of both wound types was calculated with following formula:

$$Percent\;wound\;contraction=\left(A-B\right)/A\times 100$$

where *A* is an initial wound area and *B* the area measured after a certain number of days.

#### 4.8.3. Histopathological Examination 

After 21 days, skin wound tissue of rats was excised and preserved in 10% formalin solution. Subsequently, a cross section of tissue samples was cut off from both infected and non-infected wounds and subjected to hematoxylin–eosin (H&E) reagent staining for histological examination. Photographs were captured under high resolution microscope (BX43, 40×) [38].

#### 4.8.4. Measurement of Inflammatory Mediators and Growth Factors with Real Time Polymerase Chain Reaction (RT-PCR)

On the 21st day, blood samples were drawn out in heparin vials from all treatment groups. The serum was separated out with centrifugation. Real-Time Polymerase Chain Reaction (QuantStudio3 Kits) technique was used to measure levels of inflammatory biomarkers (e.g., TNF-α) and growth factors (e.g., MMP-2 and VEGF). The mRNA was isolated and complementary DNA (cDNA) was synthesized by using cDNA Synthesis Kit (Thermo Fisher Scientific^®^, Waltham, MA, USA). Then, 2x SYBER q-PCR Master Mix was added, and samples were stored at −20 °C. Finally, mixture was kept in RT-PCR instrument (ThermoScientific^®^) [39]. Data analysis was undertaken through compilation of biological replicates and averaged. 

### 4.9. Statistical Analysis

Statistical analysis of data was performed using SPSS software (Chicago, IL, USA). Results were calculated through Post-Hoc Tuckey’s test and are presented as mean ± SEM. *p* value less than 0.05 is considered significant.

## 5. Conclusions

To summarize, AMF-*Me.mo*-loaded nanofilms have promising potential for healing wounds through the regulation of multiple molecular and cellular events (TNF-alpha, MMPs and VEGF). Moreover, the antimicrobial activity of AMF-*Me.mo*-loaded nanofilms supported the decontamination of wound sites from microbial flora, and thus can be used as a marvelous candidate for wound healing. However, long clinical trial-based studies need to be performed.

## Figures and Tables

**Table 1:** Thickness and moisture content of nanoclay-containing AMF-*Me.mo*-loaded films.

Formulation Code	Film Thickness (mm)	Moisture Content (%)
2% AMF-*Mo.me*	0.5 ± 0.02	56.4 ± 0.12
4% AMF-*Mo.me*	0.8 ± 0.01	40.5 ± 0.09
8% AMF-*Mo.me*	0.6 ± 0.01	33.6 ± 0.05

**Table 2:** Free radical scavenging of nanoclay-containing AMF-*Me.mo*-loaded films and ascorbic acid.

Formulation Code	IC_50_
2% AMF-*Mo.me*	126.20 ± 0.11
4% AMF-*Mo.me*	67.78 ± 0.07
8% AMF-*Mo.me*	68.03 ± 0.13
Ascorbic acid (standard)	27.44 ± 0.02

**Table 3:** Antibacterial effect of AMF solution, AMF-*Me.mo*-loaded nanoclay-based films (2%, 4%, and 8%), amikacin and methanolic extract of *Moringa olifera*.

*Staphylococcus aureus*						
Sr. No.	Zone of Inhibition (mm)					
	Amikacin RS	2% AMF	4% AMF	8% AMF	16% AMF	*Me.mo*
01	20.0	0.3	0.6	0.9	10.0	0.8
02	21.0	0.4	0.5	0.8	11.0	0.9
03	20.0	0.0	0.6	0.6	0.0	0.8
Average	20.3	0.2	0.6	0.8	10.5	0.8
*Escherichia coli*						
Sr. No.	Zone of inhibition (mm)					
	Amikacin RS	2% AMF	4% AMF	8% AMF	16% AMF	*Me.mo*
01	19.0	0.2	0.4	0.7	0.0	0.6
02	22.0	0.1	0.4	0.8	0.0	0.5
03	21.0	0.2	0.5	0.6	0.0	0.6
Average	20.6	0.16	0.43	0.7	0.0	0.56

RS = reference standard.

**Table 4:** Percent wound contraction in infected wounds.

Groups	3 D	7 D	10 D	14 D	21 D
Diseased Control	−76.2 ± 0.5	20.9 ± 0.1	26.1 ± 0.1	35.2 ± 0.2	25.0 ± 0.0
2% AMF-*Me.mo* Nanofilms	−46.3 ± 0.3 ***	9.1 ± 0.0 ***	17.3 ± 0.3 ***	33.3 ± 0.1 **	27.3 ± 0.0 ***
4% AMF-*Me.mo* Nanofilms	−33.2 ± 0.2v ***	10.0 ± 0.0 ***	11.1 ± 0.0 ***	37.3 ± 0.3 **	30.0 ± 0.0 ***
8% AMF-*Me.mo* Nanofilms	−33.2 ± 0.2 ***	27.5 ± 0.0 ***	17.2 ± 0.0 ***	37.5 ± 0.0 ***	73.7 ± 0.3 ***
Standard (Quench^®^)	−16.6 ± 0.1 ***	25.7 ± 0.0 ***	23.0 ± 0.0 ***	45.0 ± 0.0 ***	81.8 ± 0.1 ***

Values shown are Mean ± SEM (*n* = 4). Where ** = *p* ˂ 0.01, *** = *p* ˂ 0.001. Violet, green, and blue color stars show comparison of 2%, 4%, and 8% nanoclay-based AMF-Moringa-loaded nanofilm treatment groups with diseased control. Red color stars show comparison of standard (Quench®) with diseased control.

**Table 5:** Percent wound contraction in non-infected wounds.

Groups	3 D	7 D	10 D	14 D	21 D
Diseased Control	−70.0 ± 0.0	19.6 ± 0.0	9.7 ± 0.0	18.9 ± 0.0	33.2 ± 0.2
2% AMF-*Me.mo* Nanofilms	−56.6 ± 0.0 ***	14.9 ± 0.0 ***	15.0 ± 0.0 ***	20.6 ± 0.0 ***	22.2 ± 0.1 ***
4% AMF-*Me.mo* Nanofilms	−50.0 ± 0.0 ***	22.2 ± 0.0 ***	11.4 ± 0.0 ***	16.1 ± 0.0 **	23.1 ± 0.0 ***
8% AMF-*Me.mo* Nanofilms	−50.0 ± 0.0 ***	35.3 ± 0.3 ***	24.1 ± 0.1 ***	13.6 ± 0.0 **	21.0 ± 0.0 ***
Standard (Quench^®^)	−30.0 ± 0.0 ***	33.2 ± 0.2 ***	19.2 ± 0.0 ***	14.3 ± 0.0 ***	50.0 ± 0.0 ***

Values shown are Mean ± SEM (*n* = 4). Where ** = *p* ˂ 0.01, *** = *p* ˂ 0.001. Violet, green, and blue color stars show comparison of 2%, 4%, and 8% nanoclay-based AMF-Moringa-loaded nanofilm treatment groups with diseased control. Red color stars show comparison of standard (Quench®) with diseased control.

**Table 6:** Concentrations of pro-inflammatory and growth factors in burn wound healing model.

Groups	TNF-α	VEGF	MMP
Diseased Control	32.8 ± 0.1	32.6 ± 0.1	31.2 ± 0.1
2% AMF-*Moringa* Nanofilms	32.5 ± 0.1	32.8 ± 0.0	31.1 ± 0.1
4% AMF-*Moringa* Nanofilms	32.4 ± 0.1	33.1 ± 0.1	32.2 ± 0.1
8% AMF-*Moringa* Nanofilms	32.1 ± 0.1	33.6 ± 0.1	33.0 ± 0.0
Standard (Quench)	31.4 ± 0.0	35.2 ± 0.1	33.8 ± 0.1

Values shown are Mean ± SEM of inflammatory cytokines and growth factors in burn wound healing model, where TNF-α (tumor necrosis factor-alpha), VEGF (vascular endothelial growth factor), and MMP (metalloproteinases).

**Table 7:** Pharmaceutical composition of AMF-*Mo.me*-loaded nanoclay films.

Disc Name	Pectin (%)	Sericin (%)	Nanoclay Polymer Concentration (Phr)	Amniotic Fluid (10% of Total Polymer Weight)	*Moringa olifera* Extract (10% of Total Polymer Weight)	Glycerol (80% of Total Polymer Weight)
2% AMF-*Mo.me*	50%	50%	3	2	20	60
4% AMF-*Mo.me*	50%	50%	3	4	20	60
8% AMF-*Mo.me C*	50%	50%	3	8	20	60

## Data Availability

All data are available in this publication.
